# Preparation and Characterization of Bilayer Polymer-Dispersed Liquid Crystals Doped with Gd_2_O_3_ Nanoparticles and Rhodamine B Base Fluorescent Dye

**DOI:** 10.3390/molecules29051126

**Published:** 2024-03-02

**Authors:** Yongle Wu, Yuzhen Zhao, Xun Li, Hong Gao, Zhun Guo, Dong Wang, Yi Luan, Lei Wang

**Affiliations:** 1 Xi’an Key Laboratory of Advanced Photo-Electronics Materials and Energy Conversion Device, School of Electronic Information, Xijing University, Xi’an 710123, China; wuyongle1999@163.com (Y.W.); zyz19870226@163.com (Y.Z.); lixun1999814@163.com (X.L.); guozhun@xijing.edu.cn (Z.G.); 2 Department of Materials Physics and Chemistry, School of Materials Science and Engineering, University of Science and Technology Beijing, Beijing 100083, China; yiluan@ustb.edu.cn; 3 Division of Material Engineering, China Academy of Space Technology, Beijing 100094, China; gaohong_cast@sina.com; 4 Key Laboratory of Chemical Additives for China National Light Industry, Shaanxi Key Laboratory of Chemical Additives for Industry, College of Chemistry & Chemical Engineering, Shaanxi University of Science & Technology, Xi’an 710021, China; wanglei@sust.edu.cn

**Keywords:** polymer-dispersed liquid crystal, bilayer, electro-optical properties, Gd_2_O_3_ nanoparticles, fluorescent dyes

## Abstract

Using the polymerization-induced phase separation (PIPS) method, bilayer polymer-dispersed liquid crystal (PDLC) films with a PDLC-PVA-PDLC structure were prepared in this work. It was found that all PDLC performance indexes were affected by polymer mesh size after comparing the microscopic morphology and electro-optical properties of samples with different monomer ratios. Gd_2_O_3_ nanoparticles and rhodamine B base fluorescent dyes introduced into the bilayer PDLC optimized the samples’ electro-optical properties and developed new functionalities. In addition, the bilayer PDLC doped with Gd_2_O_3_ and rhodamine B base held excellent progressive driving functions as well as stable durability properties. Samples doped with Gd_2_O_3_ nanoparticles and rhodamine B base also produced excellent anti-counterfeiting effects under UV irradiation at different angles, further exploiting the application potential of PDLC.

## 1. Introduction

As a typical liquid crystal-based optical film, polymer-dispersed liquid crystal (PDLC) consists of a polymer substrate with embedded micrometer- or nanometer-sized liquid crystal (LC) microdroplets [1]. PDLC films exhibit an opaque state under unelectrified conditions due to the random orientation of LC microdroplets [2]. In turn, when a sufficiently large voltage is applied, LC microdroplets align themselves along the direction of the electric field and cause PDLCs to reach a transparent state with high transmittance [3]. Based on the above features, PDLC has been widely used in smart windows [4,5,6], displays [7,8], and optical switches [9,10,11]. Furthermore, PDLC is favored in low energy-consuming buildings [12,13,14], semiconductors [15], field-effect transistors [16], and solar concentrators [17].

In previous studies, functionalization and performance optimization of PDLC were achieved by optimizing polymerization conditions, adjusting precursor ratios [18,19], and doping with additional [17] special properties. Deng et al. [20] designed and prepared an ultrafast, switchable, and passive radiative cooling smart window based on PDLC that exhibited enhanced infrared emissivity in the 8–13 μm wavelength range. The novel PDLC/GO nanocomposite designed by Cheng et al. [21] responded to NIR-Vis-UV light and could be applied to soft actuators and optomechanical systems driven directly by sunlight. PDLCs accomplish fast switching speeds, large optical modulation, and robust mechanical stability using silver nanowire micromesh as electrodes, as demonstrated by Zhang et al. [22]. Shivaraja et al. [23] found that doping octadecylamine functionalized single-walled carbon nanotubes (ODA-SWCNTs) in PDLC could reduce threshold voltage and response time. The photoinitiator concentration directly affected the phase separation process, which determined the morphology and electro-optical properties of PDLCs, according to Nasir et al. [24]. In addition, other fluorescent dyes and inorganic nanoparticles were doped into PDLC for modification. Reticular nanofiber films containing the fluorescent material ST18 were blended into PDLC and improved electro-optical and fluorescent properties, according to Shi et al. [25] Electro-optical properties were enhanced by doping SiO_2_ nanoparticles into PDLC by Kim et al. [26]. Katariya-Jain et al. found that carbon nanoparticle doping could enhance thermo-electro-optical and dielectric properties in PDLC [27].

To compensate for the shortcomings of monolayer materials, multilayer [28,29] structures have become a research hotspot with applications to a variety of fields in recent years. Using a layer-by-layer method, Khoi et al. [30] deposited a polymer multi-layer membrane consisting of poly allylamine hydrochloride and poly styrene sulfonate on the cation-exchange membrane, which achieved selective recovery of Na ions. Multilayer films prepared by Culebras et al. [31] had high conductivity and Seebeck coefficients with alternately depositing layers of aqueous solutions of PEDOT: NPs and double-walled carbon nanotubes (DWNT) stabilized with PEDOT: PSS via a layer-by-layer methodology. The multilayer composite electrolyte designed and prepared by Park et al. [32] displayed excellent cyclic stability for application in lithium–sulfur batteries. The multilayer structure consisting of paper, TPS and PLA, PBAT, PHBV, or PVOH by Eslami et al. [33] not only improved the dry and wet strength of the paper but also showed considerable resistance to oil and grease. Low dielectric properties of monolayer in-plane heterostructures prevent the generation of high concentrations of thermally excited carriers from doped impurities. Ogura et al. [34] solved this problem by developing multilayer in-plane heterostructures.

However, conventional monolayer PDLC suffers from certain drawbacks, such as difficulty accessing the intermediate state between the on-state and off-state. It greatly limits the further development of PDLC. In this study, bilayer PDLC films were prepared by introducing the concept of multilayer structures into PDLC, which can access the intermediate state and achieve progressive driving functions. In addition, Gd_2_O_3_ nanoparticles and fluorescent dyes were doped into the bilayer PDLC to further improve its electro-optical properties and confer functionalization. The stable PDLC–PVA–PDLC structure also enables the bilayer PDLC to perform well in durability tests, and a longer service life can meet liquid crystal optical film application requirements.

## 2. Experimental

### 2.1. Materials

SLC1717 (n_o_ = 1.519, n_e_ = 1.720, Δn = 0.201, T_c_ = 92 °C), the nematic phase liquid crystal used in this experiment, was purchased from Shijiazhuang Chengzhi Yonghua Display Materials Co. (Shijiazhuang, China). As a mixture of UV polymerizable monomers, UV6301 was bought from Kuer Industries (Shanghai) Co. (Shanghai, China). As a cross-linker and fluorescent dye, 1,4 Butanediol dimethacrylate (BDDMA) and Rhodamine B base were purchased from Beijing Bailingwei Technology Co. (Beijing, China), respectively. The free radical photoinitiator 2,2-dimethopxy-2-phenylacetophenone (IRG651) was purchased from Anhui Zesheng Technology Co. (Anqing, China).

**Figure 1:**
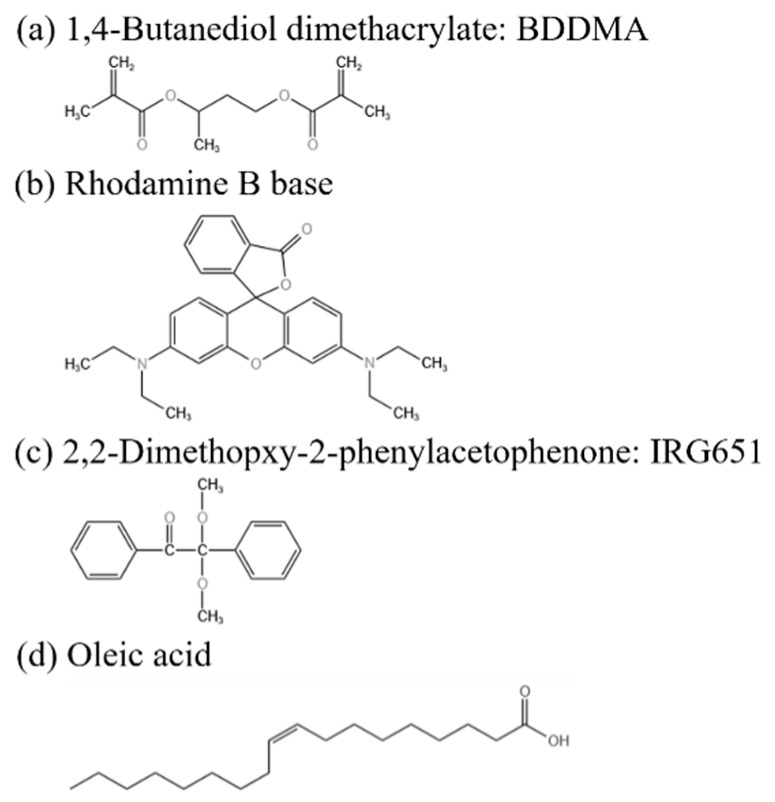
The specific chemical structure of the materials used.

Gd_2_O_3_ nanoparticles (diameter 40–60 nm), the inorganic nanoparticles used in this experiment, were acquired from Alab (Shanghai, China) Chemical Technology Co. (Shanghai, China). Oleic acid, a modifier for the nanoparticles, was bought from Sinopharm Chemical Reagent Co. (Shanghai, China). The specific chemical structures of materials used in this study are presented in Figure 1. The Gd_2_O_3_ nanoparticles were surface-modified using oleic acid to inhibit their agglomeration and uniformly disperse them in the LC/monomer mixtures.

Details concerning the surface modification treatment of Gd_2_O_3_ nanoparticles are described as follows. The Gd_2_O_3_ nanoparticles (10 mmol) were scattered in tetrahydrofuran (20 mL) and mechanically stirred (300–600 rpm) at 65 °C for 30 min for homogeneous mixing. The oleic acid (5 mL)/tetrahydrofuran (10 mL) mixture was poured into the Gd_2_O_3_ solution and continuously stirred for 30 min under heating conditions. Oleic acid (2 mL) was mixed into the Gd_2_O_3_ solution for 5 min and the procedure was repeated five times. For adequate modification, the above mixture was mechanically stirred (300–600 rpm) at 85 °C for 2 h. The heating process described above must be conducted under condensing conditions to avoid tetrahydrofuran volatilization. Afterward, the heat source was removed to cool the mixture to room temperature. The precipitate was obtained after centrifuging (5000 rpm, 5 min), separating the above blend, and washing five times with anhydrous ethanol for primary modified Gd_2_O_3_ nanoparticles. The above primary modified Gd_2_O_3_ nanoparticles were solubilized in the oleic acid (5 mL)/hexane (20 mL) mixture and mechanically stirred (800~1000 rpm) for 2 h. The precipitate was obtained after centrifuging (5000 rpm, 10 min), separating the above blend, washing five times with anhydrous ethanol, and then drying to obtain secondary modified Gd_2_O_3_ nanoparticles.

### 2.2. Sample Preparation

PVA films are ideal for preparing bilayer PDLC interlayers due to their excellent mechanical properties. In addition, PVA film is immiscible with LC, which is critical for preparing bilayer PDLC. The procedures for specific preparation are as follows.

1. Preparing precursors: LC/monomer/initiator blends in different proportions were shaken for 3 min, stirred for 5 min, and sonicated for 15 min to produce a uniform phase. Specific percentages are described in Table 1, Table 2 and Table 3;
2. Preparing monolayer PDLC: LC cells were prepared using an 8 μm thick polyimide film as a spacer to hold two single-sided conductive glass substrates apart. Mixtures a1~a6 in Table 1 were injected into LC cells by capillary action, and monolayer PDLC samples were obtained after polymerization. The polymerization time, light intensity, and temperature for this set of samples were 6 min, 4.5 mW/cm^2^, and 35 °C, respectively;
3. Preparing PVA films: A layer of aqueous PVA solution (0.05 wt%) was placed on the glass substrate with surface treatment using surface tension. Glass substrates with PVA films adhered to the surface were obtained by storing them at a temperature of 75 °C for 3 h;
4. Preparing bilayer PDLC: The ratios of various substances in the upper and lower layers of the bilayer PDLC are shown in Table 2 and Table 3, respectively. An 8 μm thick polyimide film was used to separate the glass substrate with PVA film on the surface from the unilateral conductive glass substrate and fixed to obtain the LC cell. The upper PDLC precursor was injected into the LC cell by capillary action, and PDLC-PVA composite films were obtained after curing. The glass substrates with PDLC-PVA composite film on the surface were obtained by peeling off the surface-treated glass substrates. The glass substrates attached with PDLC-PVA composite film on the surface were separated from the one-sided conductive glass substrates using 8 μm thick polyimide films to obtain new LC cells. The lower PDLC precursors were injected into new LC cells by capillary action, and bilayer PDLC films with a PDLC–PVA–PDLC structure were acquired after polymerization.

Among them, the variables of Groups B and C were the Gd_2_O_3_ nanoparticle and Rhodamine B base content, respectively, as shown in Table 2 and Table 3.

### 2.3. Characterization

To verify whether oleic acid was attached to the surface of Gd_2_O_3_ nanoparticles, its chemical composition was characterized using Fourier-Transform Infrared Spectrometry (FTIR, INVENIO S, Bruker Optics GmbH & Co. KG, Ettlingen, Germany).

Electro-optical characteristics are one of the most critical metrics and can usually measure the application potential of PDLC. In this experiment, the electro-optical curve, response time curve, and contrast ratio were evaluated with the Liquid Crystal Comprehensive Parameter Tester (LCT-5016C, Beijing LCD Engineering Research and Development Center, Beijing, China). Additionally, other vital variables such as threshold voltage (V_th_), saturation voltage (V_sat_), off-state transmittance (T_off_), and off-state response time (t_off_) were also obtained from electro-optical characterization tests. Herein, V_th_ and V_sat_ were attained once the transmittance of the bilayer PDLC reached 10% and 90% of its maximum value, respectively. The ratio of on-state to off-state transmittance is called the contrast ratio (CR).

The polymer matrix morphology was observed by scanning electron microscopy (SEM, ZEISS SUPRA55, Carl Zeiss AG, Oberkochen, Germany). Samples were pretreated prior to observation as follows: PDLC samples were immersed in cyclohexane for 15 d at room temperature to clear all LC molecules. The cyclohexane was renewed every three days during the soaking process to ensure the adequate deletion of LC molecules. After soaking, the samples were dried at 60 °C for 1 h to remove residual cyclohexane from the surface. After drying, the samples were sprayed with gold under a vacuum for SEM visualization.

The fluorescence emission spectra of bilayer PDLC samples doped with Rhodamine B base were characterized using a steady-state transient fluorescence spectrometer (Edinburgh Instruments, Livingston, UK, FLS 1000), and fluorescence emission curves were obtained for different doping concentrations.

The samples were tested for transmittance at different wavelengths with the Lambda 950 UV/VIS/NIR spectrophotometer (Perkin–Elmer, Waltham, MA, USA) at room temperature and transmittance–wavelength curves were obtained for the bilayer PDLC at different voltages.

## 3. Results and Discussions

### 3.1. Effect of the Crosslinker Content on the Property of Monolayer PDLC

**Figure 2:**
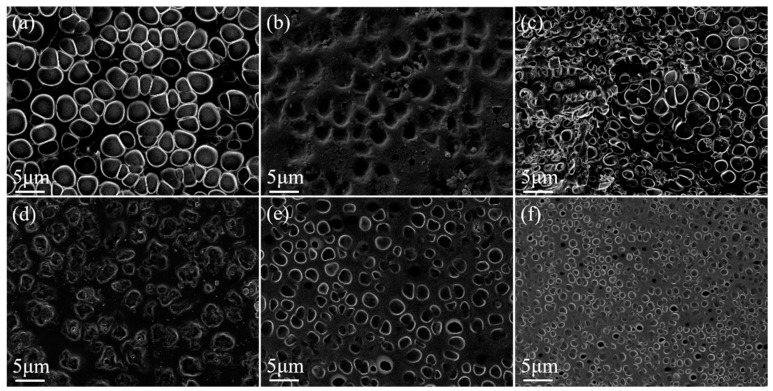
SEM images of PDLC cells. (**a**) 0 wt% BDDMA; (**b**) 1 wt% BDDMA; (**c**) 2 wt% BDDMA; (**d**) 3 wt% BDDMA; (**e**) 4 wt% BDDMA; (**f**) 5 wt% BDDMA. 3000× magnification.

Crosslinking agents connect polymer chains and reduce the size of the polymer mesh. The SEM image of the polymer mesh with variations in crosslinker BDDMA content is shown in Figure 2. As the crosslinker content increased, the number of crosslinking points in the polymer network also increased, leading to a decrease in the size of the polymer mesh.

**Figure 3:**
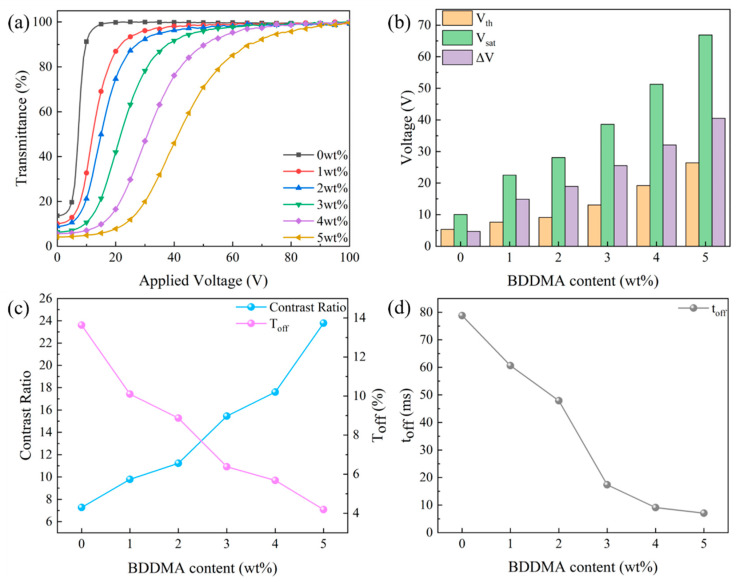
The effect of cross-linker (BDDMA) content on electro-optical properties: (**a**) voltage-transmittance curve; (**b**) threshold voltage (V_th_), saturation voltage (V_sat_), and ΔV; (**c**) contrast ratio (CR) and off-state transmittance (T_off_); (**d**) response time (t_off_).

Variations in polymer micromorphology led to changes in the electro-optical properties of PDLC, as depicted in Figure 3. The anchoring force on LC droplets increases with smaller polymer meshes, which shifts the transmittance-voltage curve to the right and raises V_th_ and V_sat_, as demonstrated in Figure 3a,b. V_th_ and V_sat_ were enlarged from 5.3 V and 10.0 V to 26.4 V and 66.9 V, respectively, when BDDMA content increased from 0 to 5 wt%. The growth of V_sat_ was greater than V_th_, increasing ΔV from 4.7 V to 40.5 V. Changes in polymer morphology can cause variation in the refractive index match between the polymer and LC microdroplets, altering the T_off_ and CR of the PDLC, as illustrated in Figure 3c. The refractive index match between the polymer and the LC microdroplets was weakened with increasing BDDMA content, causing a gradual decrease in T_off_ from 13.6% to 4.2%. CR gradually grew from 7.3 to 23.8 as the CR tendency became contrary to T_off_. The larger the anchoring force, the simpler it is for the LC microdroplets to recover from an ordered state to a disordered state. The t_off_ decreased from 78.8 ms to 7.1 ms as depicted in Figure 3d.

### 3.2. Gd_2_O_3_ Nanoparticles Doped Bilayer PDLC

#### 3.2.1. Modification of Gd_2_O_3_ Nanoparticles

**Figure 4:**
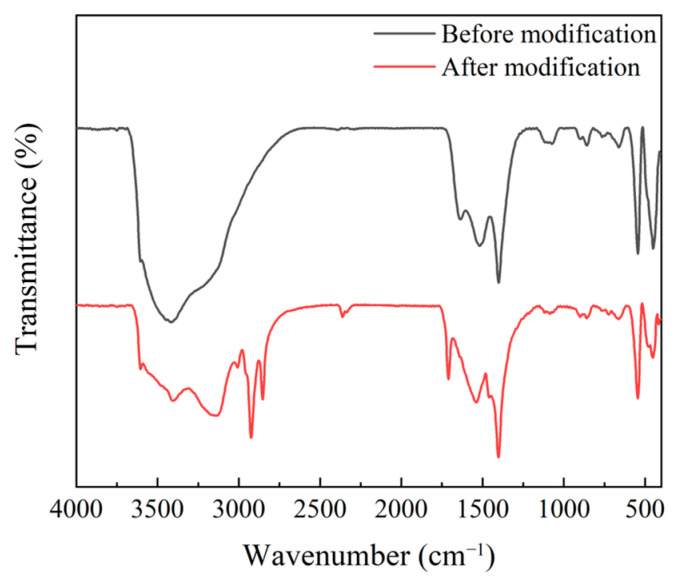
Fourier-transform infrared (FTIR) spectra of Gd_2_O_3_ nanoparticles before and after modification.

Gd_2_O_3_ nanoparticles were modified with oleic acid to prevent agglomeration and promote uniform dispersion in the precursors of LC/UV polymerizable monomers. The FTIR spectra of Gd_2_O_3_ nanoparticles before and after modification are shown in Figure 4. Compared to pre-modification, the modified Gd_2_O_3_ nanoparticles showed a characteristic peak at 2900 cm^−1^, which represents the stretching vibration peak of the methylene group. It can be seen that oleic acid was successfully modified on the surface of Gd_2_O_3_ nanoparticles.

#### 3.2.2. Effect of Gd_2_O_3_ Nanoparticle Content on the Properties of Bilayer PDLC

**Figure 5:**
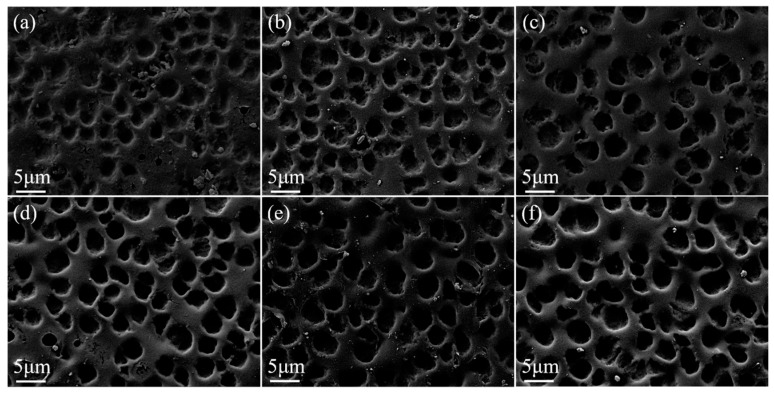
SEM images of upper-layer PDLC cells. (**a**) 0.0 wt%; (**b**) 0.2 wt%; (**c**) 0.4 wt%; (**d**) 0.6 wt%; (**e**) 0.8 wt%; (**f**) 1.0 wt% Gd_2_O_3_ nanoparticles doped. 3000× magnification.

**Figure 6:**
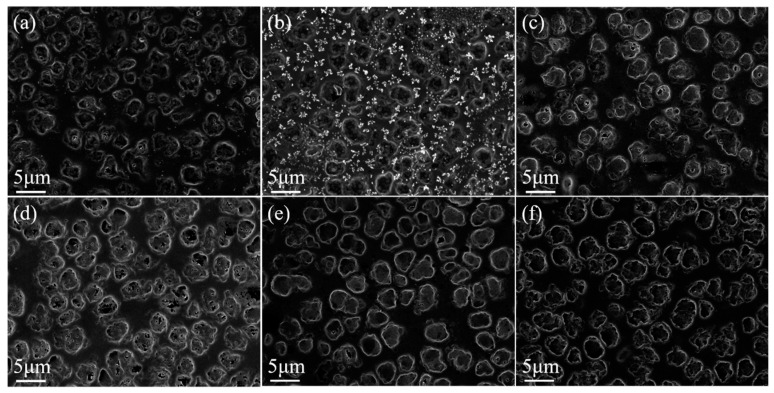
SEM images of lower-layer PDLC cells. (**a**) 0.0 wt%; (**b**) 0.2 wt%; (**c**) 0.4 wt%; (**d**) 0.6 wt%; (**e**) 0.8 wt%; (**f**) 1.0 wt% Gd_2_O_3_ nanoparticles doped; 3000× magnification.

Gd_2_O_3_ nanoparticles affect polymers’ microscopic morphology, as indicated in Figure 5 and Figure 6. Gd_2_O_3_ nanoparticles could access LC microdroplets and increase their size, thus changing the polymer’s micromorphology. As Gd_2_O_3_ nanoparticle content increased, the size of LC microdroplets also increased, resulting in a gradual increase in polymer mesh size.

**Figure 7:**
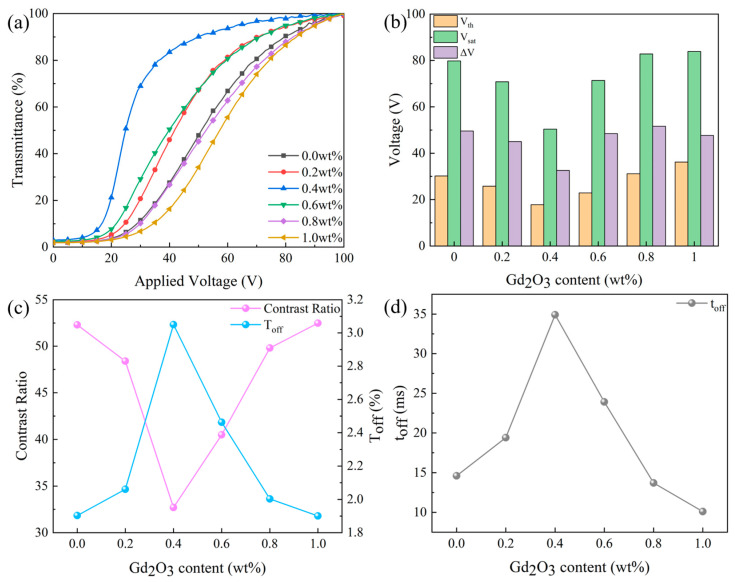
The effect of Gd_2_O_3_ nanoparticle content on electro-optical properties. (**a**) voltage-transmittance curve; (**b**) threshold voltage (V_th_), saturation voltage (V_sat_), and ΔV; (**c**) contrast ratio (CR) and off-state transmittance (T_off_); (**d**) response time (t_off_).

As shown in Figure 7, the fraction of Gd_2_O_3_ nanoparticles significantly impacts the electro-optical properties of the bilayer PDLC. Electro-optical curves migrated to the right and then left as the size of the polymer mesh increased in Figure 7a. While the percentage of Gd_2_O_3_ nanoparticles enlarged from 0 to 0.4 wt%, the anchoring force applied to the LC droplets progressively declined, bringing about reductions in V_th_ and V_sat_ of the bilayer PDLC from 30.2 V and 79.8 V to 17.8 V and 50.4 V, respectively. Since the reduced value of V_sat_ was greater than V_th_, ΔV decreased, according to Figure 7b. With the increase in Gd_2_O_3_ nanoparticles to 1 wt%, V_th_ and V_sat_ gradually increased to 36.2 V and 83.9 V, while ΔV was roughly kept at 50 V.

Additionally, the fraction of Gd_2_O_3_ nanoparticles impacted the T_off_ and CR of bilayer PDLC, as demonstrated in Figure 7c. The increase in Gd_2_O_3_ nanoparticle content from 0 to 0.4 wt% improved the T_off_ from 1.9% to 3.0%. However, a further increase in Gd_2_O_3_ nanoparticle content to 1.0 wt% brought about a decrease in T_off_ to 1.9%. Given that the CR’s tendency was the opposite of T_off_, the CR first decreased from 52.3 to 32.71 and then increased to 52.5. Variations in Gd_2_O_3_ nanoparticle content affected the time required for LC droplets to transition from an ordered to a disordered state, as observed in Figure 7d. By increasing the Gd_2_O_3_ nanoparticle content from 0 to 0.4 wt%, the t_off_ increased from 14.6 ms to 34.9 ms. However, as the nanoparticle content was further extended to 1.0 wt%, the t_off_ decreased to 10.1 ms.

**Figure 8:**
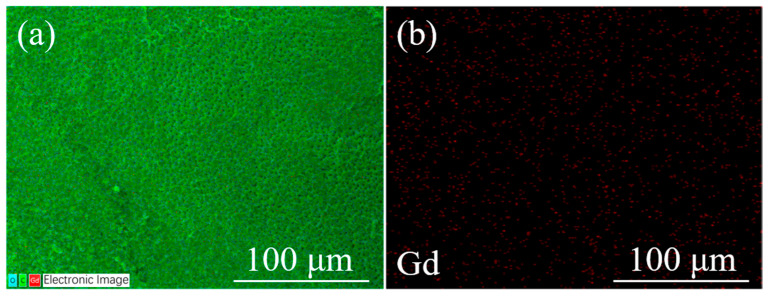
EDS mapping of the optimal sample (b2). (**a**) The distribution of all elements and (**b**) the distribution of Gd elements.

The EDS map of the sample with the optimal Gd_2_O_3_ nanoparticle content (b2) is shown in Figure 8. According to the Gd elemental distribution, the Gd_2_O_3_ nanoparticles are more uniformly distributed. In addition, the agglomeration of Gd_2_O_3_ nanoparticles is not obvious, which is a prerequisite for maintaining PDLC’s excellent performance.

#### 3.2.3. Progressive Driving Test of Bilayer PDLC Doped with Gd_2_O_3_ Nanoparticles

**Figure 9:**
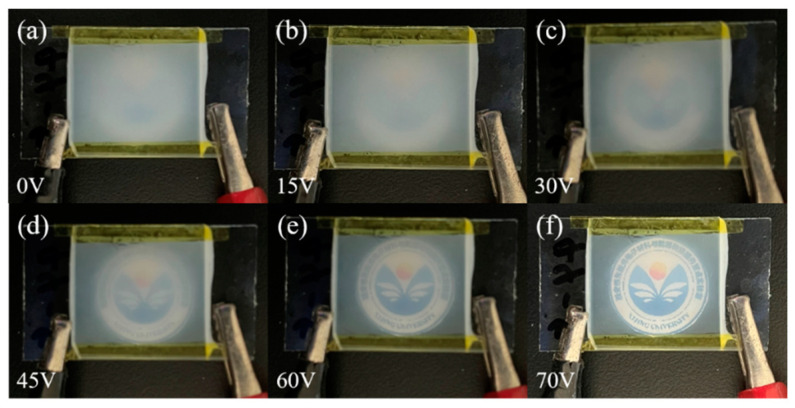
Physical plots of transmittance variation for the optimum sample at different voltages: (**a**) 0 V, (**b**) 15 V, (**c**) 30 V, (**d**) 45 V, (**e**) 60 V, and (**f**) 70 V, respectively.

**Figure 10:**
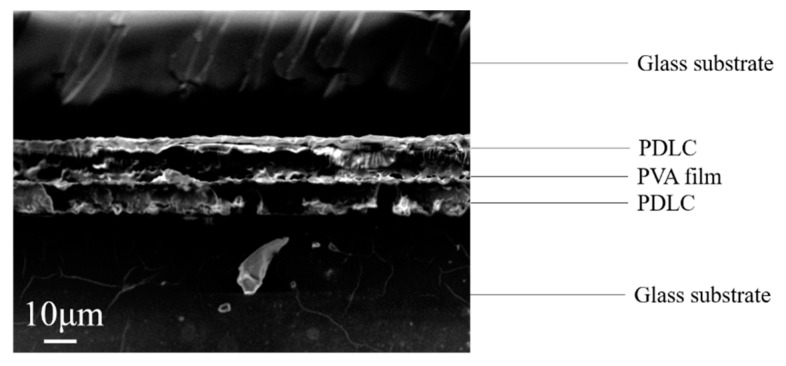
SEM image of a bilayer PDLC cross-section with a PDLC–PVA–PDLC structure.

The change in transmittance was observed by applying different voltages to the sample and verifying the progressive driving performance of the bilayer PDLC. As the applied voltage increased, there was a stepwise growth in the sample transmittance, as shown in the physical diagram in Figure 9. The reason for this result is that the two monolayer PDLC samples have different drive voltages so both cannot be driven simultaneously. The cross-sectional SEM image of the bilayer PDLC is plotted in Figure 10, where one layer of the PDLC is easier to drive and the other is more difficult to drive.

### 3.3. Fluorescent Dye Rhodamine B Base Doped Bilayer PDLC

#### 3.3.1. Effect of the Fluorescent Dye Rhodamine B Base on Bilayer PDLC Properties

**Figure 11:**
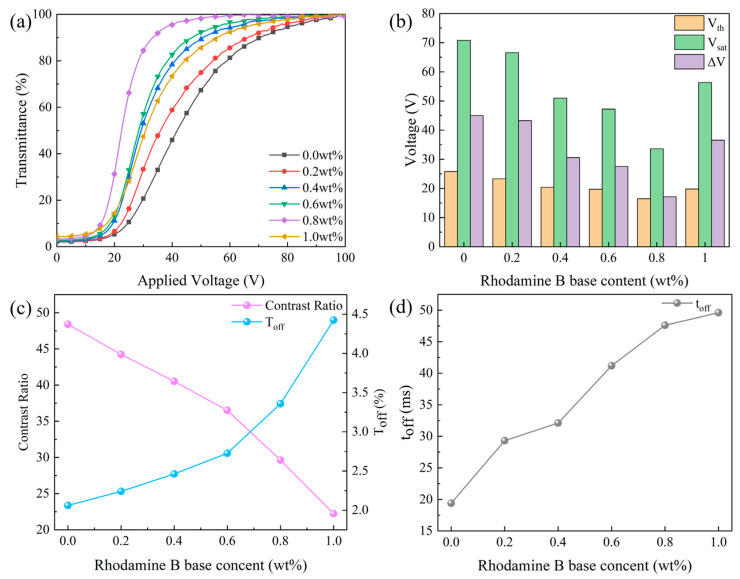
The effect of Rhodamine B base content on electro-optical properties. (**a**) Voltage-transmittance curve; (**b**) threshold voltage (V_th_), saturation voltage (V_sat_), and ΔV; (**c**) contrast ratio (CR) and off-state transmittance (T_off_); (**d**) response time (t_off_).

In the set of experiments, different rhodamine B base levels were incorporated into the precursors to explore the influence of different rhodamine B base levels on bilayer PDLC performance. The impact of rhodamine B base contention on the electro-optical properties of bilayer PDLC is summarized in Figure 11. Fluorescent dyes can produce fluorescent effects and absorb UV light under UV irradiation, inducing a diminution of light radiation intensity in polymerized monomers. Consequently, the polymerization rate reduces and leads to an expansion of the polymer mesh and LC droplet sizes.

After the addition of Rhodamine B base proportion from 0 to 0.8 wt%, the transmittance-voltage curve moved to the left and caused a gradual reduction in V_th_ and V_sat_ from 25.8 V and 70.8 V to 16.5 V and 33.6 V. With the further increase in rhodamine B base content to 1.0 wt%, the transmittance–voltage curves moved to the right and V_th_ and V_sat_ increased to 19.8 V and 56.3 V, as illustrated in Figure 11a,b. The reason for this result is that as the fluorescent dye content increased, the anchoring force on the LC droplets decreased and became easier to drive. However, too much fluorescent dye content led to poor conductivity in the sample, which caused the driving voltage to increase.

Rhodamine B base content also affected the CR of bilayer PDLC, as depicted in Figure 11c. Since T_off_ gradually decreased with increasing rhodamine B base content, the CR gradually increased. The fluorescent dye increased the refractive index match between the polymer network and LC microdroplets, which increased the T_off_ of the bilayer PDLC and decreased the CR. In addition, rhodamine B base content affected the response time of bilayer PDLC, according to Figure 11d. As the anchoring force on LC microdroplets gradually decreased, the time required for their transition from the ordered to the disordered state increased, leading to an increase in t_off_.

**Figure 12:**
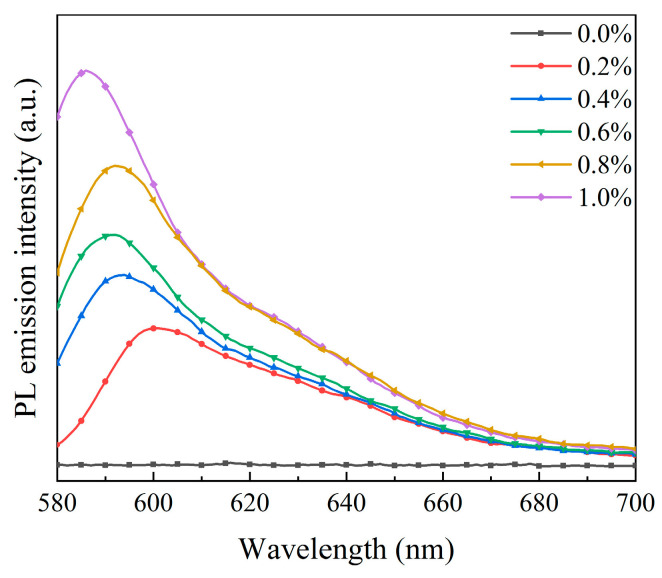
Fluorescence emission spectra of bilayer PDLC doped with rhodamine B base.

**Figure 13:**
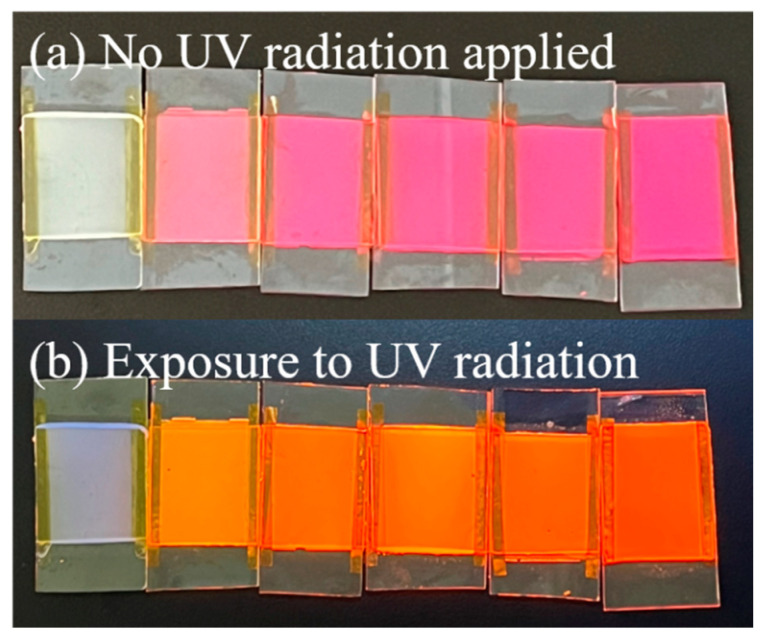
Physical image of bilayer PDLC doped with rhodamine B base. (**a**) No UV radiation applied, (**b**) Exposure to UV radiation.

The fluorescence emission spectra and visual pictures of bilayer PDLC samples doped with different rhodamine B base percentages are depicted in Figure 12 and Figure 13, respectively. There was a marked upward trend in the fluorescence emission intensity of bilayer PDLC samples as the rhodamine B base content increased. Additionally, there was a series of emission peaks within the metering range, positioned at 590 nm. Compared to no UV irradiation, the samples’ orange coloration becomes more pronounced with the increase in rhodamine B base levels, as shown in the physical diagram in Figure 13. One explanation for this is that adding rhodamine B base to PDLC caused a strong fluorescence intensity, which was enhanced by increased fluorescent dye content.

#### 3.3.2. Progressive Driving Test of Bilayer PDLC Doped with Rhodamine B Base

**Figure 14:**
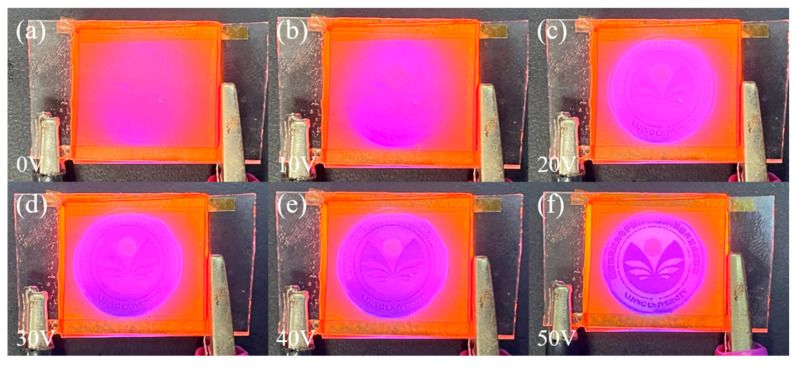
Physical plots of transmittance variation of the optimum sample doped with Rhodamine B base at different voltages: (**a**) 0 V, (**b**) 10 V, (**c**) 20 V, (**d**) 30 V, (**e**) 40 V, and (**f**) 50 V, respectively.

The physical plots of the transmittance variation of bilayer PDLC doped with rhodamine B base under UV irradiation at different voltages are demonstrated in Figure 14. With the increase of voltage, the transmittance of the bilayer PDLC increases gradually and the pattern at the bottom is clearer, which indicates that the bilayer PDLC sample has favorable progressive driving performance. In addition, due to the fluorescence effect of rhodamine B base, the color of the pattern through the sample changed accordingly.

### 3.4. High Temperature and Strong UV Light Radiation Tests

**Figure 15:**
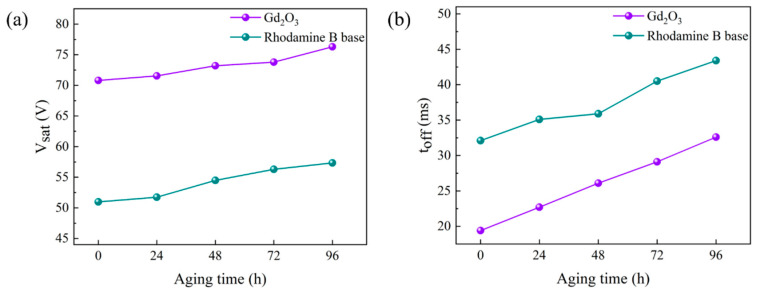
Effects of high temperature and strong ultraviolet radiation on (**a**) saturation voltage (V_sat_) and (**b**) response time (t_off_).

The prepared bilayer PDLC samples b2 and c3 were tested for aging resistance in a long-term high temperature (50 °C) and strong UV (830 mW/cm^2^) environment. The test samples were subjected to multiple tests at high temperatures and under UV light to test their electro-optical properties. Specific changes in V_sat_ and t_off_ are shown in Figure 15, which both increase with aging time. The rate of change in samples doped with rhodamine B base was less than the undoped ones, suggesting that doping with rhodamine B base helps PDLC performance under extreme conditions and has a better lifetime. The presence of rhodamine B base absorbs UV light and converts it to orange light, thereby reducing the damage caused by UV light to the polymer network and providing endless possibilities for future developments.

### 3.5. Application of Bilayer PDLC Doped with Rhodamine B Base in Multi-Angle Anti-Counterfeiting

**Figure 16:**
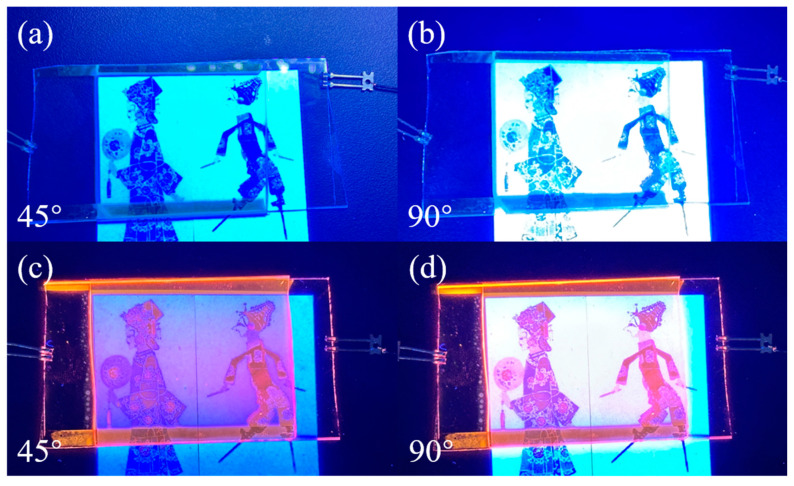
Application of bilayer PDLC in anti-counterfeiting. (**a**) Undoped rhodamine B base and UV 45° oblique light; (**b**) undoped rhodamine B base and UV 90° direct light; (**c**) doped rhodamine B base and UV 45° oblique light; (**d**) doped rhodamine B base and UV 90° direct light.

Bilayer PDLC samples doped with rhodamine B base showed orange coloration by fluorescence emission under UV light irradiation. Setting a specific color at a specific location in the pattern can reveal another color when superimposed with the orange color emitted by the fluorescence of Rhodamine B. Using this feature, PDLC samples doped with rhodamine B base can be applied to the field of anti-counterfeiting. When voltage is applied, the pattern underneath the sample will show a special light after changing the angle of UV irradiation, as shown in Figure 16. When undoped with Rhodamine B, the pattern color is displayed normally. However, when doped with Rhodamine B, special colors develop at specific locations in the pattern. Using UV light to illuminate the sample at different angles will also have different color effects.

## 4. Conclusions

In conclusion, many problems in conventional PDLC devices, such as difficulty accessing the intermediate state between the on-state and the off-state under low-voltage conditions, can be solved by introducing Gd_2_O_3_ nanoparticles and rhodamine B base. According to our experimental results, the polymer network was affected by the ratio of LC/polymerizable monomer, which led to changes in electro-optical properties. In addition, the binding of Gd_2_O_3_ to LC microdroplets and the fluorescence emission from rhodamine B base also affected the polymer mesh, thus conferring excellent electro-optical properties on bilayer PDLCs. Compared to the original samples, samples obtained with Gd_2_O_3_ and rhodamine B base at the optimal content had superior electro-optical properties, with V_th_ and V_sat_ reduced by about 30% and 35%, respectively. The multi-angle anti-counterfeiting function can also be practiced by the doping of rhodamine B base, leading to innovative application areas for PDLC. Doping with Gd_2_O_3_ nanoparticles and fluorescent dye rhodamine B base not only improved electro-optical properties but also the functionalization of PDLC films. This study provides promising new ideas for preparing functional PDLC devices.

## Figures and Tables

**Table 1:** The composition of Group A ^a^.

Sample	Weight Percentage (wt%)		
	SLC1717	UV6301	BDDMA
Group A			
a1	50	50	0
a2	50	49	1
a3	50	48	2
a4	50	47	3
a5	50	46	4
a6	50	45	5

^a^ The weight of the photo-initiator IRG651 is 0.5% of the total weight.

**Table 2:** The upper compositions of Groups B and C.

Sample	Weight Percentage (wt%)				
	SLC1717	UV6301	BDDMA	Gd_2_O_3_ Nanoparticles	Rhodamine B Base
Group B					
b1	50	49	1	0.0	0.0
b2	50	49	1	0.2	0.0
b3	50	49	1	0.4	0.0
b4	50	49	1	0.6	0.0
b4	50	49	1	0.8	0.0
b6	50	49	1	1.0	0.0
Group C					
c1	50	49	1	0.2	0.0
c2	50	49	1	0.2	0.2
c3	50	49	1	0.2	0.4
c4	50	49	1	0.2	0.6
c5	50	49	1	0.2	0.8
c6	50	49	1	0.2	1.0

**Table 3:** The lower composition of Groups B and C.

Sample	Weight Percentage (wt%)				
	SLC1717	UV6301	BDDMA	Gd_2_O_3_ Nanoparticles	Rhodamine B Base
Group B					
b1	50	47	3	0.0	0.0
b2	50	47	3	0.2	0.0
b3	50	47	3	0.4	0.0
b4	50	47	3	0.6	0.0
b4	50	47	3	0.8	0.0
b6	50	47	3	1.0	0.0
Group C					
c1	50	47	3	0.2	0.0
c2	50	47	3	0.2	0.2
c3	50	47	3	0.2	0.4
c4	50	47	3	0.2	0.6
c5	50	47	3	0.2	0.8
c6	50	47	3	0.2	1.0

## Data Availability

The data presented in this study are available in article.
